# Glycogen storage disease type VII (Tarui disease): a case report presenting a *PFKM* variant previously described only in canine models

**DOI:** 10.3389/fgene.2026.1925751

**Published:** 2026-09-01

**Authors:** Mariapia Griffo, Nicola Molitierno, Laura Napoli, Michela Ripolone, Simona Zanotti, Francesco Fortunato, Gabriele Tumminello, Maurizio Moggio, Marco Locatelli, Stefania Paola Corti, Giacomo Pietro Comi, Dario Ronchi

**Affiliations:** 1 Department of Pathophysiology and Transplantation, Dino Ferrari Center, University of Milan, Milan, Italy; 2 Neuromuscular and Rare Diseases Unit, Fondazione IRCCS Ca’ Granda Ospedale Maggiore Policlinico, Milan, Italy; 3 Department of Cardio-Thoracic-Vascular Diseases, Fondazione IRCCS Ca’ Granda Ospedale Maggiore, Policlinico, Italy; 4 Unit of Neurosurgery, Fondazione IRCCS Ca’ Granda Ospedale Maggiore, Policlinico, Italy; 5 Department of Medical-Surgical Physiopathology and Transplantation, University of Milan, Milan, Italy; 6 Neurology Unit, Fondazione IRCCS Ca’ Granda Ospedale Maggiore Policlinico, Milan, Italy

**Keywords:** glycogen storage disease, GSD-VII, metabolic myopathy, PFKM, phosphofructokinase, Tarui disease

## Abstract

Glycogen storage disease type VII (GSD-VII), or Tarui disease, is a rare autosomal recessive disorder caused by biallelic loss-of-function variants in the *PFKM* gene encoding the muscle isoform of phosphofructokinase (PFK), a key enzyme of the glycolytic pathway. PFK deficiency impairs glycogen and glucose metabolism in skeletal muscle and erythrocytes, causing exercise intolerance, exertional myalgia, and myoglobinuria, and, in some cases, fixed proximal muscle weakness, as well as haemolytic anaemia. We report the case of an Italian woman with genetically confirmed GSD-VII harbouring a homozygous missense variant in *PFKM* (NM_000289.6:c.550C>T, p.Arg184Trp). This variant was previously identified in Wachtelhund dogs, a spontaneous animal model of PFK deficiency, but never reported in patients so far. PFK activity in skeletal muscle (PFKM) was found severely decreased and ultrastructural analysis revealed glycogen accumulation and mitochondrial alteration, supporting the pathogenetic role of the identified variant.

## Introduction

Glycogen storage disease type VII (GSD-VII; OMIM #232800) or Tarui disease is a rare autosomal recessive metabolic myopathy due to biallelic pathogenic variants in the *PFKM* gene (12q13.11) encoding the muscle isoform of phosphofructokinase (PFK; EC 2.7.1.11). Phosphofructokinase catalyses the phosphorylation of fructose-6-phosphate to fructose-1,6-bisphosphate, a rate-limiting step of the glycolytic cascade. Tissue specific isoforms of phosphofructokinase have been also found in liver (L-isoform) and platelets/fibroblasts (P-isoform) and independently encoded by *PFKL* (21q22.3) and *PFKP* (10p15.2) genes, respectively (Howard et al., 1996).

In skeletal muscle, PFK assembles into a tetramer of M subunits, whereas in erythrocytes, PFK exists as a mixture of five tetrameric isozymes (M4, M3L, M2L2, ML3, L4) generated by random combination of M and L subunits (Vora et al., 1980).

This explains why a lack of PFKM protein abolishes PFK enzyme activity completely in muscle but only partially reduces it in red blood cells, where L subunit provides residual compensation for PFK deficiency. Thus, it is evident why GSD-VII presents predominantly myopathic manifestations, given that non-muscle tissues maintain partial PFK function because of tissue-specific isoforms’ expression (Toscano and Musumeci, 2007; Auranen et al., 2015).

The disease was first described by Tarui and collaborators in 1965, in a report of three affected Japanese siblings presenting with exercise intolerance, muscle weakness, and elevated creatine kinase (CK) activity (Tarui et al., 1965). Since then, more than 100 patients with GSD-VII have been reported worldwide, with a disproportionately higher prevalence in individuals of Ashkenazi Jewish origin (Sherman et al., 1994; Raben and Sherman, 1995).

Four main phenotypes can be distinguished in GSD-VII: 1) childhood-onset type with exercise-related cramps, myoglobinuria, and compensated hemolytic anemia; 2) late-onset type with fixed proximal muscle weakness and no prominent exertional symptoms; 3) infantile type with hypotonia, progressive myopathy, cardiomyopathy, and early respiratory failure; 4) hemolytic type without overt muscle disease.

To date, less than 30 pathogenic variants (Chen et al., 2024) in the *PFKM* gene have been described, including deletions, duplications, insertions, and single nucleotide changes. Diagnosis is based on clinical manifestations, demonstration of decreased PFK activity in muscle or erythrocytes, and molecular confirmation by direct or next-generation sequencing (NGS) analysis of *PFKM* coding regions. Muscle biopsy with histochemical staining of PFK enzyme activity was initially considered a gold-standard method for diagnosing the disease, but this approach may provide false-negative results (Auranen et al., 2015).

PFK deficiency is a well-documented condition in veterinary medicine, occurring in English Springer Spaniels, American Cocker Spaniels, Whippets, and Wachtelhunds, presenting with hemolytic anemia and exertional myopathy. A missense variant at c.550C>T p. (Arg184Trp) in the canine *PFKM* gene was identified as responsible for the disease in Wachtelhunds (Inal Gultekin et al., 2012). Identification of the identical variant in a human patient, as described in the present report, represents an unprecedented observation in the literature.

## Case report

**FIGURE 1:**
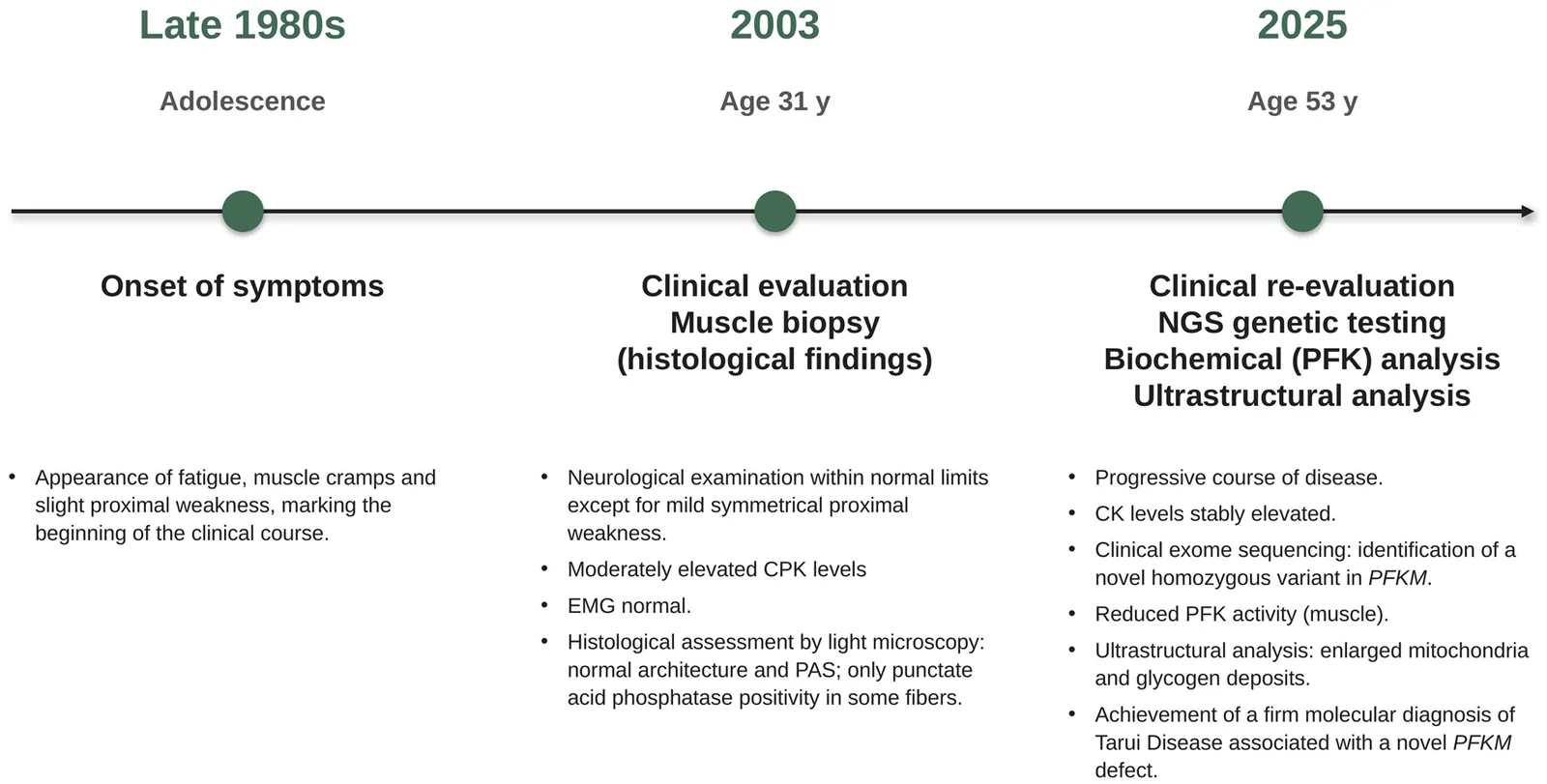
Patient’s timeline. Main clinical events in patient’s clinical history.

A 53-year-old Italian woman was referred to our Neuromuscular and Rare Diseases Unit for evaluation of persistent myalgia, easy fatigability, and exertional cramps that had been present since adolescence and gradually progressed over decades. Her symptoms worsened after prolonged walking, and she reported occasional gait unsteadiness. There was no history of myoglobinuria or dark urine. Cardiac involvement was excluded by standard electrocardiogram and echocardiography. Main clinical events in patient’s clinical history are available in Patient’s timeline (Figure 1).

Patient’s parents, clinically unaffected, are first cousins. Patient’s sister was diagnosed with cognitive and motor developmental delay while her brother displayed intellectual disability.

Family members did not provide consent to undergo clinical and genetic investigations, and consequently no additional information could be obtained. Parental consanguinity prompted suspicion of a potential autosomal recessive disorder in the patient.

General physical examination was unremarkable. Neurological examination showed only mild symmetrical proximal muscle weakness (Medical Research Council (MRC) grade 4+/5) in her upper limbs (deltoids, biceps, and triceps muscles) and lower limbs (iliopsoas, quadriceps, and hamstrings). Bulbar and ocular functions were intact. Reflexes were symmetrical and preserved. Sensation was intact. Skin examination did not reveal rash or joint hypermobility, and there was no dysmorphism.

Common blood test panels, including complete blood cell count, liver and kidney function tests, thyroid function, and inflammatory markers, were within normal ranges. Hemolytic anemia was excluded based on normal bilirubin, haptoglobin, and reticulocyte count. Direct measurement of erythrocyte PFK enzymatic activity was not performed given that molecular confirmation had been prioritised in the diagnostic workup, and erythrocyte PFK activity testing is not universally available in the context of a late-onset phenotype without overt haemolysis. Serial CK measurements demonstrated persistently elevated values above the upper limit of normal (ULN), with exacerbation following physical exertion (most recent value 900 U/L; peak value up to 8,000 U/L). Lactate dehydrogenase (LDH) was occasionally elevated.

Electromyography (EMG) including nerve conduction studies was unremarkable, with no signs of myotonia, abnormal spontaneous activity, or myopathic motor unit changes.

**FIGURE 2:**
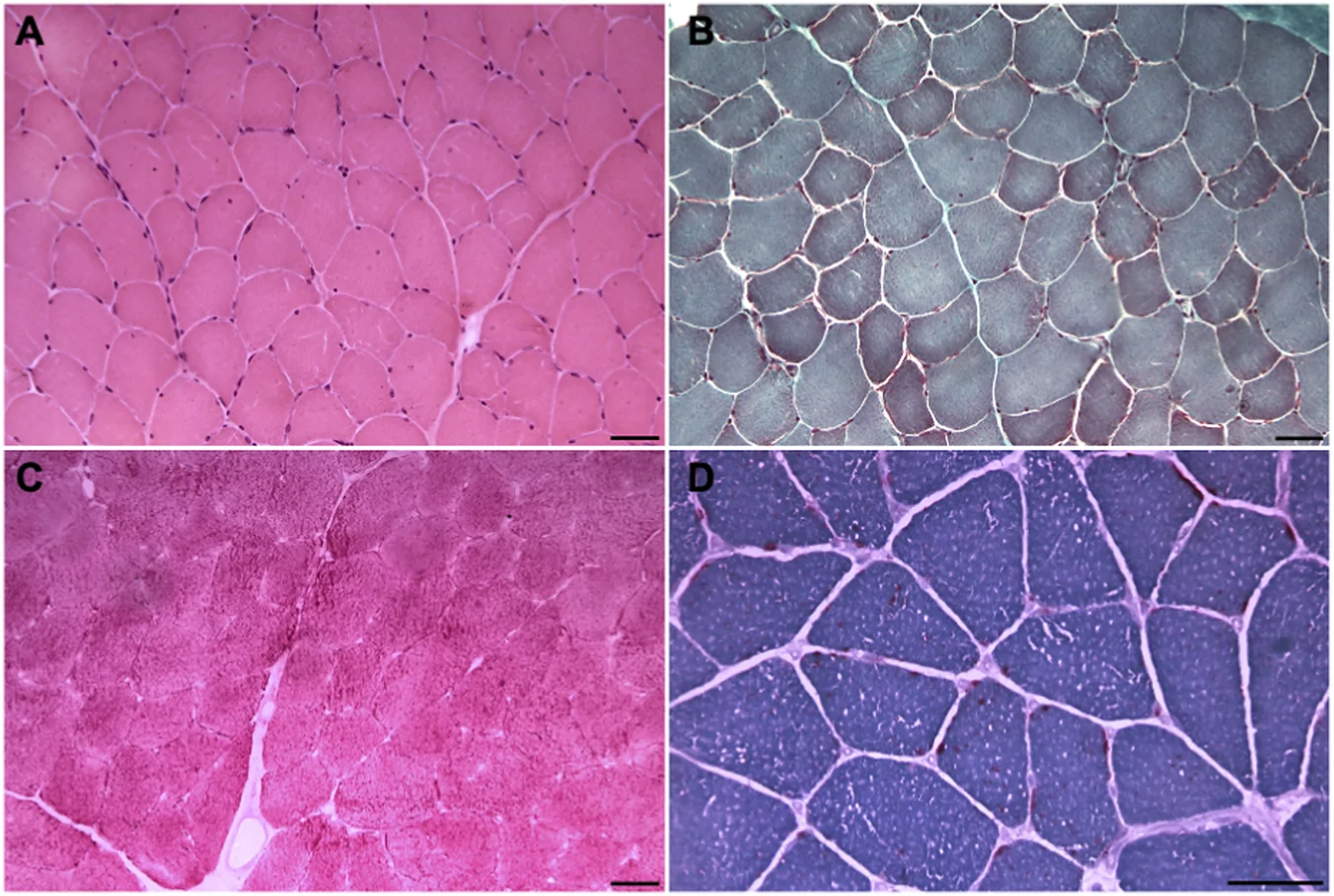
Muscle biopsy: histological findings. **(A)** Hematoxylin and Eosin (H&E) and **(B)** modified Gomori trichrome (MGT) staining revealed preserved skeletal muscle architecture, with myofibers of relatively homogeneous size, no evident inflammatory infiltrates or necrotic fibers, and no abnormal increase in connective tissue or inclusions. **(C)** PAS staining demonstrated normal glycogen distribution within myofibers. **(D)** Acid phosphatase staining showed intracytoplasmic punctate positivity in a subset of myofibers. Scale bar 20 µm.

Histopathological examination of the muscle biopsy (deltoid muscle, performed at 31 years of age) showed preserved skeletal muscle architecture. Myofibers appeared of relatively homogeneous size, with no evident inflammatory infiltrates, necrotic fibers, abnormal increase in connective tissue, or inclusions (Figures 2A,B). PAS staining confirmed a normal distribution of glycogen within the myofibers (Figure 2C), whereas acid phosphatase staining revealed intracytoplasmic punctate positivity in a subset of fibers (Figure 2D).

The gradual clinical worsening and the persistence of elevated CK values prompted a re-evaluation of the case in our Neuromuscular unit.

**FIGURE 3:**
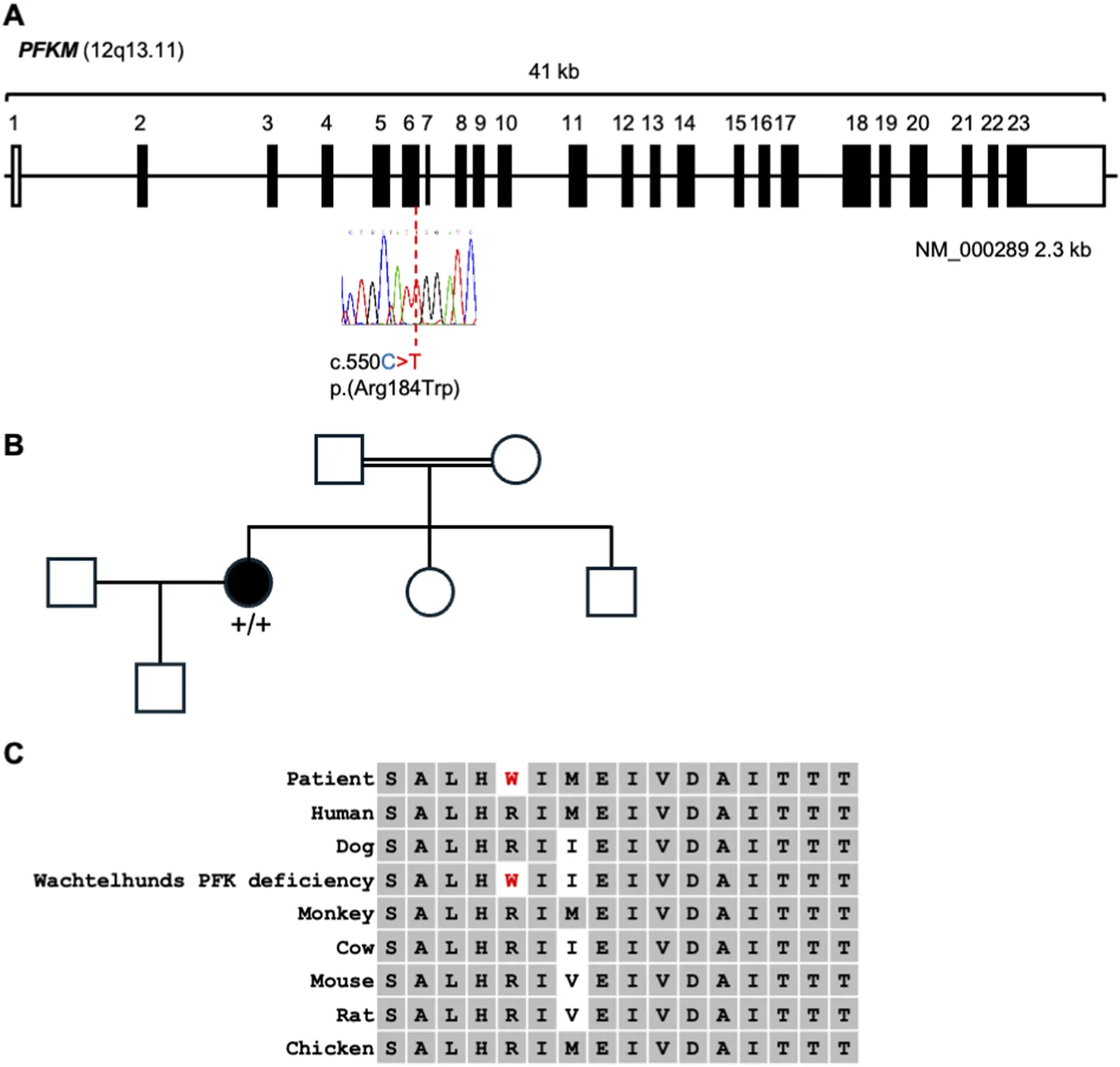
Molecular findings. **(A)** Genomic organization of *PFKM* (12q13.11; RefSeq NM_000289; 23 exons spanning 41 kb); filled boxes represent coding exons and open boxes the untranslated regions. The Sanger electropherogram shows the homozygous c.550C>T transition, predicted to result in the missense substitution p.(Arg184Trp); the affected nucleotide is marked by the dashed red line. **(B)** Pedigree of the family. Filled symbol: affected proband; open symbols: unaffected members; squares: males; circles: females; double horizontal line: parental consanguinity. Homozygosity for the c.550C>T variant in the patient is indicated with “+/+”. **(C)** Conservation of amino acid positions surrounding the affected codon (p.Arg184, R) which is replaced by a tryptophan (W) in the patient and in a canine model of PFK deficiency.

Clinical exome sequencing followed by variant prioritization on a virtual panel of genes involved in metabolic myopathies revealed the rare (gnomAD MAF: 0.000682, number of homozygotes: 0) homozygous c.550C>T variant in exon 6 (NM_000289.6) of *PFKM* gene likely resulting in the p. (Arg184Trp) aminoacid change (Figure 3A).

Several computational pathogenicity prediction tools suggested that this variant was deleterious/probably damaging (REVEL score: 0.842). Segregation analysis was not possible owing to the unavailability of the patient’s parents’ DNA samples. However, the homozygous status in the context of consanguineous parents is suggestive of identity by descent (Figure 3B). The variant was classified as of unknown significance.

A literature search revealed that the identical nucleotide substitution, c.550C>T leading to p. (Arg184Trp) change, had been identified in the orthologous canine *PFKM* gene in Wachtelhund dogs with hereditary PFK deficiency (Inal Gultekin et al., 2012). In the canine transcript used by Inal Gultekin et al. (NM_001003199.1, expressed in blood), this variant is located in exon 8; however, that transcript lacks two upstream exons that are present in the testis-expressed canine isoform. When aligned to the human transcript numbering (NM_000289.6), the equivalent variant resides in exon 6. This difference reflects transcript-level annotation rather than a true species-specific exon structure. The substituted amino acid residue Arg184 resides within a helical domain of the PFKM protein and is highly conserved across vertebrate species (Figure 3C). Conservation of the residue and the demonstrated pathogenicity of the variant in the orthologous species support a pathogenic role in the human setting.

The availability of the muscle biopsy allowed the execution of biochemical and ultrastructural analysis to obtain novel findings supporting the pathogenic role of the identified variant.

Biochemical analysis of PFK activity performed in the skeletal muscle biopsy showed dramatic decrease of residual activity: 0.02 μmol/min/mg of protein compared to normal range of 0.91±0.21 μmol/min/mg. Glucose-6-phosphate isomerase (GPI) activity, used as a reference, did not show differences between the patient (1.91 μmol/min/mg of protein) and the controls (2.22±0.60 μmol/min/mg). The other glycolytic enzymes were normal as well (Supplementary Figure S1).

**FIGURE 4:**
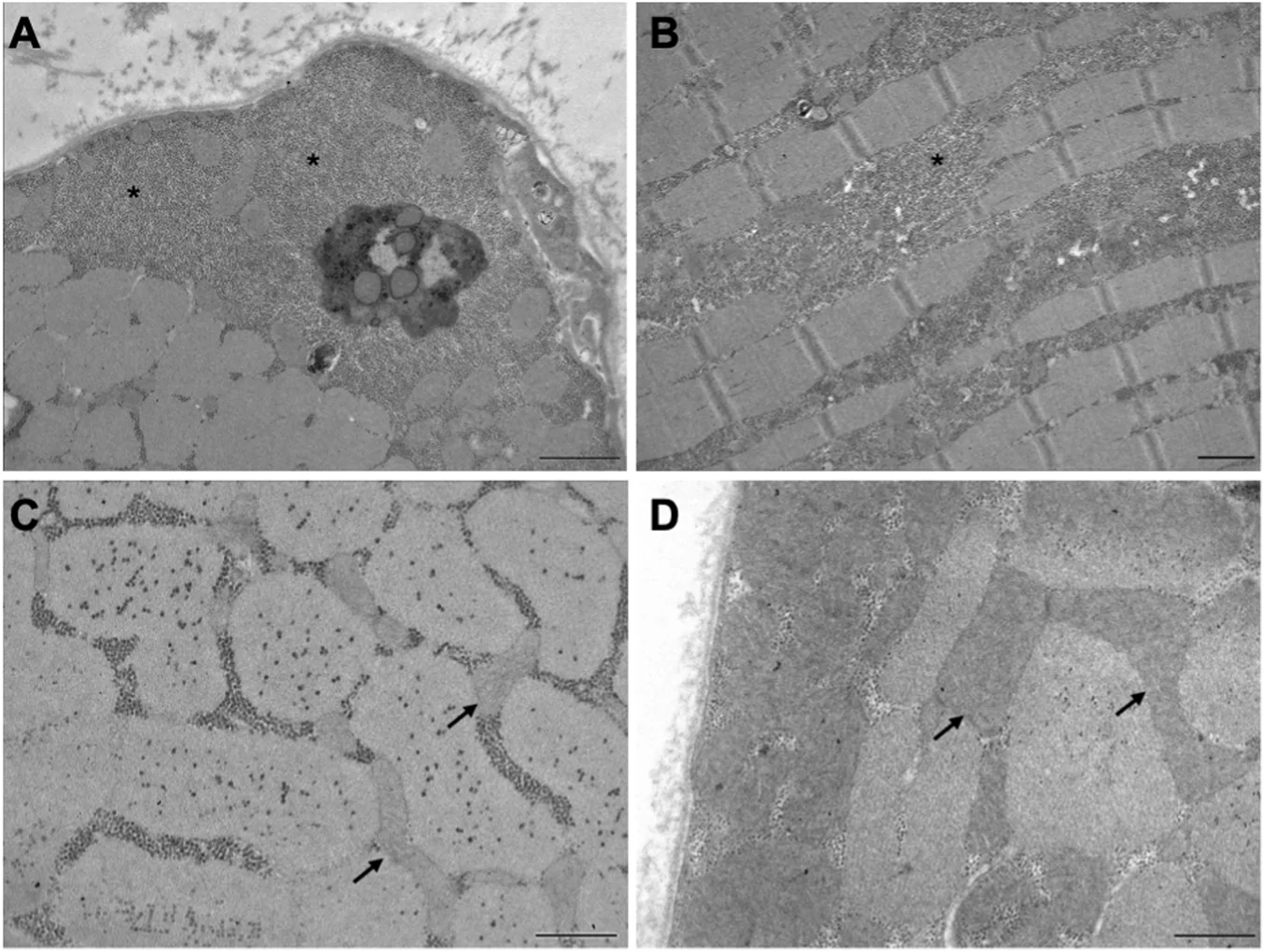
Muscle biopsy: ultrastructural analysis. **(A)** Marked accumulation of free glycogen localized in the subsarcolemmal region, **(B)** glycogen detected within the intermyofibrillar and intramyofibrillar compartments, **(C,D)** enlarged mitochondria. Asterisks indicate glycogen deposits. Arrows indicate enlarged mitochondria. Bar: (A,B) 1 μm; (C,D) 0.5 µm.

Ultrastructural analysis revealed a marked accumulation of free glycogen, predominantly localized in the subsarcolemmal region, with additional deposits detected within the intermyofibrillar and intramyofibrillar compartments. Enlarged mitochondria were also observed, while preserving normal cristae architecture and matrix morphology. In addition, numerous lipofuscin granules were identified, almost consistently in a subsarcolemmal distribution and frequently associated with glycogen deposits (Figure 4).

Applying ACMG/AMP criteria, the homozygous VUS was reclassified as likely pathogenic (PS3, PM2, PP3_moderate).

Based on clinical, biochemical, and genetic findings, the diagnosis of Tarui disease (GSD-VII) was established. The patient was enrolled in a follow-up clinical monitoring program.

Reaching a definitive diagnosis after decades of unexplained myalgia and fatigability was reported as a relief for the patient, providing an explanation for her symptoms. The diagnosis also allowed practical dietary and behavioural recommendations to be provided, aimed at reducing the frequency of exertional myalgia episodes. Furthermore, the patient was informed of the potential value of extending genetic screening to other family members, however, she and her relatives chose not to pursue this option. This decision was respected, in recognition of the sensitive and personal nature of genetic testing within the family.

## Discussion

This report presents the first human patient with GSD-VII carrying a homozygous *PFKM* variant p.(Arg184Trp) so far found only in Wachtelhund dogs.

The clinical phenotype of the patient corresponds to a late-onset subtype of GSD-VII, with fixed mild proximal muscle weakness and exertional myalgia without recurrent myoglobinuria or hemolysis. The absence of hemolytic anemia in the patient may be explained by preserved PFK activity in erythrocytes, where L subunit isoforms partially compensate for the M subunit deficiency (Vora et al., 1980; Toscano and Musumeci, 2007).

Regarding the molecular aspects, Arg184 is located within an alpha helix of the PFKM protein and is highly conserved across vertebrates (Figure 3C). Substitution of arginine with the bulkier and hydrophobic amino acid tryptophan was hypothesised to alter the local protein structure (Inal Gultekin et al., 2012). The occurrence of the identical variant in two evolutionarily distant species with overlapping phenotypic manifestations strongly supports its pathogenicity.

The documented parental consanguinity and the homozygous variant status are consistent with an autosomal recessive inheritance pattern and identity by descent. The cognitive and motor disabilities observed in the patient’s siblings may reflect independent genetic or perinatal causes; nonetheless, molecular genetic evaluation of the wider family would be warranted.

The histological pattern on light microscopy in GSD-VII is classically described as characterised by non-rimmed subsarcolemmal vacuoles with extralysosomal glycogen accumulation detectable by PAS staining, and in some cases, by a fraction of diastase-resistant PAS-positive material corresponding to polyglucosan bodies (Servidei et al., 1986; DiMauro and Spiegel, 2011; Malfatti et al., 2012), possibly reflecting the accumulation of glucose-6-phosphate upstream of the glycolytic block (Vorgerd et al., 1996). However, the extent of these alterations can vary significantly. Muscle biopsies in GSD VII often reveal only a mild increase in subsarcolemmal glycogen, with the overall muscle architecture appearing largely preserved; furthermore, total muscle glycogen content is not markedly elevated, often appearing only slightly increased or at the upper limit of normal, with a normal distribution pattern (Lucia et al., 2021). In our patient, the histological findings performed at the age of 31 years showed largely preserved muscle architecture and no significant abnormalities.

The recent re-evaluation of the case in our center, followed by genetic testing with the identification of the homozygous c.550C>T p. (Arg184Trp) *PFKM* variant, prompted the ultrastructural analysis of the muscle biopsy which revealed marked glycogen accumulation within subsarcolemmal, intermyofibrillar, and intramyofibrillar compartments. The punctate acid phosphatase positivity and the subsarcolemmal lipofuscin granules likely represent a secondary autophagic-lysosomal activation related to chronic glycogen storage, in line with the lysosomal and mitochondrial changes previously reported in late-onset PFK deficiency (Sivakumar et al., 1996). The mild morphological phenotype is consistent with the missense variant p.(Arg184Trp), which preserves residual activity, in contrast with the more severe forms caused by truncating variants. This was confirmed by biochemical analysis of phosphofructokinase activity in muscle tissue which conclusively demonstrated PFK deficiency.

This case highlights how the complementary use of genetic analysis, enzymatic biochemistry, and electron microscopy may lead to definitive diagnosis, particularly in cases where conventional histology is not conclusive.

The clinical diagnosis of GSD-VII is challenging even for experienced physicians. The phenotypic spectrum has progressively widened, and adult cases with long-standing disease may develop hypertrophic cardiomyopathy and paroxysmal atrial fibrillation (Musumeci et al., 2012), justifying cardiological follow-up in patients with prolonged history. Because *PFKM* shows wide allelic heterogeneity without consistent genotype-phenotype correlations, the diagnostic process still requires the integration of clinical, biochemical, histological, and molecular data (Musumeci et al., 2012).

GSD-VII belongs to the clinically and etiologically heterogeneous group of metabolic myopathies whose diagnosis is often complicated by overlapping clinical symptoms such as recurrent myoglobinuria and exercise intolerance which can be observed in glycogen storage diseases, fatty acid oxidation defects, and mitochondrial disorders due to respiratory chain impairment (Tarnopolsky, 2022). Nevertheless, distinct clinical manifestations might help to discriminate these conditions. For example, Tarui (GSD VII) and McArdle (GSD V) diseases are almost clinically identical forms of muscle glycogenosis. McArdle patients display the second wind phenomenon, often aggravated by a carbohydrate load. PFK deficiency also carries extra-muscular signs lacking in McArdle disease such as compensated hemolytic anemia, although our case testifies that this aspect may not be present. Although clinical and biochemical clues might help to differentiate metabolic myopathies (Berardo et al., 2010), genetic studies should be prioritized to achieve a fast and reliable diagnosis of these conditions.

The absence of overt EMG abnormalities, limited information obtained from muscle biopsy, and variable CK activity are characteristic features of this disease and may cause delayed diagnosis. In a patient with exertional myalgia, consanguineous parents, and episodic CK elevation, genetic testing should be considered as an initial diagnostic tool rather than relying solely on functional testing of enzyme activity or muscle histochemistry.

Forearm exercise testing, muscle MRI, and respiratory function tests were not performed in the patient described, representing limitations of the present report.

Forearm exercise testing, typically used as a first-line functional screening tool, and muscle MRI were not performed as the diagnostic workup was primarily oriented toward molecular and biochemical confirmation once the biopsy material became available. Respiratory function tests were not performed, consistent with the absence of clinical signs suggestive of respiratory muscle involvement and in line with the mild, late-onset phenotype observed in this patient. These functional and imaging assessments should 2 be considered in future prospective evaluations of similar patients.

No specific treatment for GSD-VII is currently available, and management relies primarily on lifestyle modifications. Avoidance of high-intensity anaerobic exercise is recommended. Carbohydrate-rich meals immediately before exertion should also be avoided, as glucose availability paradoxically worsens exercise tolerance in this condition by suppressing lipolysis and thereby depriving skeletal muscle of fatty acid substrates (Haller and Lewis, 1991). A low-carbohydrate diet and regular moderate aerobic exercise may be beneficial (Lucia et al., 2021).

A prudent pharmacological approach is essential, since previous reports have highlighted that specific drugs may worsen the underlying clinical condition (Filosto et al., 2019).

In the study of GSD-VII, a cross-species approach could be particularly informative. Knockout mouse models of PFK deficiency have already been generated, and the identification of the same missense variant in dogs raises the interesting possibility of using naturally occurring canine models as a complementary tool for research. Naturally occurring animal models of this kind can add value alongside rodent data, since they sometimes reflect features of the human phenotype that mice fail to capture (Inal Gultekin et al., 2012; García et al., 2009).

## Conclusion

We describe here the first human case of GSD-VII associated with a homozygous *PFKM* variant p.(Arg184Trp) previously documented only in Wachtelhund dogs. This report bridges veterinary and human genetics, provides compelling cross-species evidence for the pathogenicity of this variant, and expands the mutational spectrum of Tarui disease. Cross-species variant annotation represents a promising complementary strategy in variant classification for rare Mendelian disorders.

## Data Availability

The datasets presented in this article are not readily available because they contain information that could compromise the privacy of the research participant. Requests to access the datasets should be directed to the corresponding author (dario.ronchi@unimi.it).
