# Supplementary material for: Glycogen storage disease type VII (Tarui disease): a case report presenting a PFKM variant previously described only in canine models
Source: Front Genet. 2026 Sep 1;17:1925751. doi: 10.3389/fgene.2026.1925751 (PMC13574603; doi:10.3389/fgene.2026.1925751)
Supplement: Supplementary file 1 [file Image1.pdf]

Supplementary Figure S1. Biochemical analysis of glycolytic enzyme activities in the patient's skeletal muscle biopsy compared with control values.

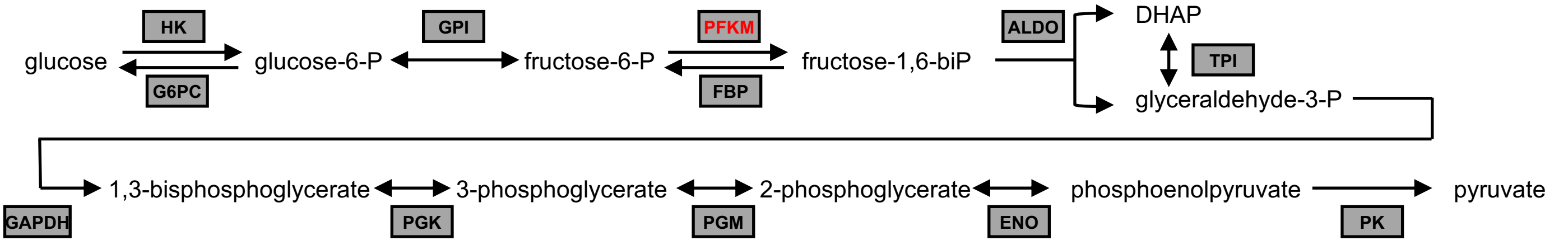

Enzyme	Patient	Controls (n=20, mean ± standard deviation)
Glucose-6-phosphate isomerase (GPI)	1.91	2.22 ± 0.60
Phosphofructokinase (PFKM)	**0.02**	**0.91 ± 0.21**
Aldolase (ALDO)	0.54	0.65 ± 0.14
Glyceraldehyde-3-phosphate dehydrogenase (GAPDH)	2.21	2.98 ± 0.85
Phosphoglycerate kinase (PGK)	1.23	1.53 ± 0.36
Phosphoglycerate mutase (PGM)	2.80	4.29 ± 1.67
Enolase (ENO)	1.23	1.43 ± 0.42
Pyruvate kinase (PK)	2.93	2.23 ± 0.47
Lactate dehydrogenase (LDH)	2.13	2.38 ± 0.79
